# Co-creation in research, policy engagement and advocacy: Lessons and limits from two decades of practice at the African Population and Health Research Center

**DOI:** 10.1371/journal.pgph.0007405

**Published:** 2026-09-30

**Authors:** Emmanuel Oloche Otukpa, Abdhalah K. Ziraba, Luchuo Engelbert Bain

**Affiliations:** 1 International programs, African Population and Health Research Center (APHRC) Campus, Nairobi, Kenya; 2 Health Systems Strengthening Hub, Institute of Healthcare Management, Strathmore University, Nairobi, Kenya; 3 Nuffield Department of Women’s & Reproductive Health (NDWRH), University of Oxford, Oxford, United Kingdom,; 4 Faculty of Humanities, Department of Psychology, University of Johannesburg, Johannesburg, South Africa; NYU Grossman School of Medicine: New York University School of Medicine, UNITED STATES OF AMERICA

## Abstract

Co-creation is widely advocated in global health research and inconsistently practiced. This paper offers a reflective, practice-based synthesis of two decades of co-created research, policy engagement and advocacy at the African Population and Health Research Center (APHRC).We define co-creation through four dimensions; shared agenda setting, joint knowledge production, power sharing, and community leadership and ownership. We distinguish it from consultation, stakeholder engagement and partnership, and assess each dimension on a graded scale. To this we add a second axis: the reach of co-creation across the research-to-policy-to-advocacy interface, and the condition of the transitions between its segments. Six cases spanning evidence generation, policy processes, capacity strengthening and rights-based advocacy are assessed against both axes. Three findings emerge. Co-creation in this institution’s practice is substantive where knowledge is produced and weak where authority is held. Agendas were substantively shared in four of six cases and financial resources in none. It fails at the transitions between research, policy engagement and advocacy rather than within them, at moments that were neither designed nor evaluated nor recorded. And the domains of co-creation differ systematically in the dimensions they can realize, with community leadership substantive only in rights-based advocacy. We draw practice guidance on governance, funding, community roles, transitions and assessment, and state what a single-institution insider synthesis cannot establish.

## Introduction

The concept of co-creation when applied to research, policy engagement, and advocacy directed towards health systems strengthening and global health is a collaborative paradigm that brings together diverse actors, including researchers, policymakers, and community members, to address complex health challenges and improve health outcomes [1]. Its appeal rests on the claim that integrating local knowledge and perspectives, particularly from marginalized communities (residents of informal settlements, vulnerable adolescents and sexual and gender minorities), produces that interventions are more relevant, acceptable and sustainable. The term now circulates widely in global health, and its diffusion has outpaced agreement on what it means. It is used interchangeably with consultation, with partnership and with stakeholder engagement, and applied to work in which the distribution of authority is entirely conventional.

This paper examines two decades of co-created work at the African Population and Health Research Center (APHRC), a pan-African research and capacity-strengthening institution headquartered in Nairobi and asks a narrower question than the literature usually poses: not whether co-creation works, but how much of it was present, across how much of the work, and what determined the answer.

### A working definition with four dimensions

This reflexive analysis depends on the precision of its central concept, as such, we adopt an explicit working definition. We understand co-creation as a collaborative mode of producing research, policy engagement, and advocacy in which non-academic actors including policymakers, practitioners, and community members participate as genuine co-producers rather than as subjects, informants, or beneficiaries [2]. So defined, co-creation is distinct from, though related to, adjacent practices that lie along a continuum of participation: *consultation and stakeholder engagement*, in which actors are informed or canvassed for input; and partnership or collaboration, in which actors share resources or jointly execute activities but may retain separate agendas and distinct point of authority. Advocacy, similarly, is a goal or activity that may or may not be co-created, rather than a synonym for co-creation. Co-creation is the more demanding subset of collaborative practice in which actors share in defining the problem, in decision-making, and in ownership of the resulting knowledge and outputs [3]. We operationalize this definition through four dimensions that we apply consistently across the cases that follow: *shared agenda setting, joint knowledge production, power sharing, and community leadership and ownership*. We treat co-creation as a matter of degree, and we regard it as effective where these dimensions are substantively realized and where the process yields demonstrable results across the research-to-policy-to-advocacy interface, such as the formulation or uptake of policy, the building of durable capacity, or the conduct of legitimate, community-owned advocacy.

A definition of this kind earns its place only if it can classify an actual project. Table 1 attempts to clarify; who defines the problem, who holds decision rights, who owns the outputs and what happens when the parties disagree. The last of these is the sharpest discriminator, and we state it as a single test that we apply to every case: could the non-academic actors have changed the direction of the work against the researchers’ preference, and is there evidence that they did? Consultation and stakeholder engagement fail this test by design; collaboration answers it separately for each partner within its own sphere; co-creation requires that the answer be yes across the shared work.

**Table 1:** Co-creation distinguished from adjacent practices.

Practice	Who defines the problem	Who holds decision rights	Who owns outputs	What happens when the parties disagree
Consultation	The research team; input is invited on a question already set	The team, entirely	The team	Disagreement is recorded; it need not change anything
Stakeholder engagement	The team; actors are mapped and engaged to secure relevance or uptake	The team, conceding influence selectively	The team; shared for use	Disagreement is managed, and often absorbed
Collaboration/ partnership	Jointly or in parallel, by parties with separate mandates	Each party, over its own contribution	Jointly authored, separately owned	Negotiated between principals; either party may withdraw
Co-creation	Jointly, from the outset	Shared, including over what the work is for	Jointly owned, with non-academic actors able to determine use	Resolved through an agreed process in which non-academic actors can prevail

### Co-creation, co-production and co-design: The right terminology

The vocabulary of this field is unsettled, and because our argument turns on definitional precision, we state where our usage sits. Three distinct literatures converge on the prefix. The first, originating in Ostrom’s analysis of public services, treats co-production as the involvement of citizens in producing the services they consume, and is concerned principally with efficiency, legitimacy and the boundary between state and citizen [4]. The second, associated with science and technology studies, uses the observation that how a society knows the world and how it governs the world are made together [5]. The third, descending from Scandinavian participatory design, treats co-design and co-creation as methods for distributing design authority to those affected by what is designed [6,7]. Health research draws on all three, often without distinguishing them, which is one reason the terms circulate as near-synonyms in practice [8]

Our usage is closest to the third, methodological tradition, and we retain the term co-creation for that reason. The second is significant to our argument, since it explains why the question of whose knowledge counts is constitutive of what gets produced rather than a procedural detail of how it is produced [5,9]. And the first supplies a caution we take seriously throughout: participation can be mobilized to legitimate decisions already taken, or to transfer cost and labor rather than authority [10]. We do not treat co-creation, co-production and co-design as synonyms, and where we use the neighboring terms we mean them in these senses.

### Co-creation as a matter of degree

We treat co-creation as a matter of degree in two respects: in intensity, meaning the extent to which each dimension is substantively realized rather than nominally observed; and in extent, meaning how far co-creation is sustained across the research-to-policy-to-advocacy interface rather than confined to one segment of it. We regard co-creation as effective where the dimensions are substantively realized, where the practice is sustained across the transitions between segments rather than lapsing at them, and where the process yields demonstrable results the formulation or uptake of policy, the building of durable capacity, or the conduct of legitimate, community-owned advocacy.

This treatment is not itself a novel claim. It restates for research what Arnstein proposed for citizen participation more than five decades ago that participation without redistributed power is an empty ritual [11] and it shares Cornwall’s insistence that participation names a range of practices whose differences matter more than their shared label [12]. Our contribution lies not in asserting that co-creation admits of degree but in giving that gradation an operational form: four dimensions and two axes against which cases can be scored, contested and compared.

### Contribution, attribution and what is not claimed

This paper makes four contributions. The first is operational: we specify four dimensions of co-creation and assess each on a graded scale, which converts the widely repeated claim that co-creation is a matter of degree into something that can be applied to a particular initiative, defended and contested. The second is conceptual: we introduce a second axis, the reach of co-creation across the research-to-policy-to-advocacy interface and argue that the transitions between its segments are analytically distinct from the segments themselves. The third is empirical: applying both axes to six cases drawn from three decades of one institution’s practice, we find that co-creation is substantive where knowledge is produced and weak where authority is held, and that it lapses at the transitions rather than within them. The fourth is reflexive: we offer a worked example of insider institutional analysis in global health, in which an African-led research institution is examined as at once a product of the asymmetries it addresses and an agent working against them.

We are explicit about what we do not claim. We do not claim to demonstrate that co-creation works; our design documents contribution and plausible pathways and cannot support causal attribution. We do not propose a new theory of co-creation, and we relate our account throughout to existing traditions. And we do not generalize from six purposively selected cases within a single institution: the lessons we draw are offered as transferable principles and as propositions for testing, not as findings about co-creation in general.

## Co-creation at the APHRC: Institutional context and analytical framework

To properly engage with the cases of co-creation in the subsequent section we layout certain pre-requisites. The institutions in which they occurred, the frameworks they operationalize, the practical means by which co-creation is conducted, as well as the basis on which effectiveness can be assessed.

### The African Population and Health Research Center

APHRC provides an instructive setting in which to examine these dynamics [13]. The Center began in 2001, as a research fellowship program and was subsequently constituted as an independent institution; over more than two decades it has grown into a pan-African research and capacity-strengthening hub headquartered in Nairobi, Kenya [14]. The Center’s origins lie within the question about who produces knowledge concerning Africa. In 1995 the Population Council established, in Nairobi, a population policy research fellowship programme; the Africa Population Policy Research Centre. with support from the Rockefeller Foundation [14]. The programme’s premise was that African researchers were marginal to the scientific discourse about African populations, that this marginality reflected the distribution of opportunity, training and institutional infrastructure rather than any distribution of capability, and that a research center based on the continent and staffed by African researchers was the appropriate remedy. Its early leadership, recruited from the diaspora and from within the region, was brought in on that explicit understanding, and the programme’s stated objective was to build an African institution capable of producing population and health research of a standard that would be read internationally and used domestically [15].

Between 2000 and 2001 the programme was constituted as an autonomous institution, registered in the United States, with a headquarters agreement with the Government of Kenya, and separated its finances from the Population Council; it began independent operation in 2001. Independence was neither automatic nor secure. The Center’s early independent operation depended heavily on a small number of philanthropic funders, and the withdrawal of one of them within a few years placed its continuation in question until a sustained round of competitive proposals rebuilt the funding base. We record this because it bears on an argument we make later: institutional ownership of the kind that permits genuine co-creation is expensive, contested and rarely conferred, and where it exists it has generally had to be constructed [16].

Its current mandate spans the generation of rigorous evidence on population and public health, the strengthening of research capacity across SSA, and sustained policy engagement and advocacy [14,17]. These functions are organized through a dedicated Policy Engagement and Advocacy unit and an International Programs division, and are complemented by capacity-strengthening platforms including the African Doctoral Dissertation Research Fellowship (ADDRF), the Consortium for Advanced Research Training in Africa (CARTA), the APHRC Virtual Academy, and Africa Research Connect [14,17–20]. Operationally, the Center treats systems thinking as a standard in program planning and implementation, embeds co-creation and tailored policy-engagement strategies across its work, and applies an evidence-to-action model to translate research into policy and practice [21]. Its principal thematic research areas include; health and wellbeing, human development, population dynamics and urbanization, policy engagement as well as, research and related capacity strengthening [14].

The doctoral training programmes described later arose from a diagnosis of the same kind that prompted the Center’s own founding. In recruiting researchers to staff a growing portfolio, the Center encountered a systematic gap between the preparation of doctoral graduates trained in the region and that of their counterparts trained elsewhere in research design, in methodological training and in the capacity to defend work independently which it attributed to the conditions of doctoral education rather than to the candidates. The ADDRF and CARTA were designed as responses to that diagnosis and built to work through existing African universities rather than around them [18,19,22].

We examine APHRC not as a neutral exemplar but as an institution that is at once a product and a driver of co-creation. It is a product in the sense that its own structure and capacity were themselves co-produced: the Center grew from a fellowship program into a continental hub through co-created models of doctoral training and research networking [14]. It is a driver in the sense that it actively constructs and sustains the interface between research, policy engagement, and advocacy. Convening diverse actors, building shared platforms, setting collaborative agendas, and channeling co-produced evidence into advocacy [14,17–20]. Viewing APHRC through this dual lens allows us to treat the research–policy–advocacy interface not as a fixed backdrop but as a dynamic that co-creation actively mediates, through joint problem framing, shared ownership of evidence, the embedding of policy and community actors in research design, and the feedback of advocacy into research agendas. This dual position also implicates the authors of this paper, and we return to what it means for the reliability of our account in the discussion section.

### Underlying frameworks

Co-creation in research, policy engagement, and advocacy for global health is largely informed by a range of contemporary theories. Three of which bear on the work described here. These theories aim to enhance the understanding of the steps and processes that facilitate improved collaboration between various stakeholders, beyond just the researchers, policymakers, including community actors and target research populations. Each carries a signature analytic move that can be identified in practice, and it is the presence of that move, rather than the invocation of the framework’s name, that we look for in the cases.

#### Participatory design.

Co-creation is often framed within participatory design approaches that leverage the experience and knowledge of non-academic actors. This approach recognizes that the actors, have valuable insights that can inform research and policy-making processes [1,2]. Engaging these voices ensures that interventions are relevant and effective in meeting the needs of the populations they are intended to serve. Participatory design originated in Scandinavian workplace research in the 1970s and 1980s, where its animating commitment was explicitly political: that those whose work would be reshaped by a technology had a right to determine its design, and that their situated knowledge was a condition of designing well rather than a courtesy extended to them [23,24]. Its later diffusion into service and health contexts brought the method into settings where the users are patients, communities and publics, and generated the neighboring vocabularies of co-design and co-creation [25,26].

In health research the tradition arrived principally through community-based participatory research, which established both the methods and the ethical commitments now widely assumed: that communities participate as partners across all phases rather than as sites of data collection, that findings return to those who generated them, and that capacity and benefit are shared [27,28]. That literature is also candid about the demands involved the time required, the difficulty of sustaining partnership across a project’s life, and the persistent risk that participation is insignificant [29].

The signature analytic move is the transfer of specified design or interpretive decisions to non-academic actors at defined stages, and it is the transfer, rather than the consultation preceding it, that distinguishes participatory design from stakeholder engagement. Bødker and Kyng have argued that the field’s diffusion has come at the cost of precisely this commitment, with participatory methods retained while the redistribution of authority that motivated them is quietly dropped [23]. That warning applies directly to co-creation in global health, and we return to it in assessing our own cases.

#### Systems thinking.

A key theoretical framework around co-creation is the *systems thinking* [30]. This framework advocates for the assessment of all critical sub-systems that affect decision-making and service delivery at multiple levels; including political, social, and economic factors [30,31]. With the adoption of a systems thinking perspective, stakeholders can better understand the complexities, interconnectedness and the interdependence among various actors within and without the healthcare system, which is important for successful co-creation efforts.

Systems thinking entered health systems research as a corrective to interventions designed as if they acted on isolated components rather than within an interdependent whole [32]. Its central propositions are that system behavior arises from the relationships between elements rather than from the elements themselves, that feedback and delay make effects non-linear and often counter-intuitive, and that interventions therefore produce consequences at points remote from where they are applied [32,33]. For health policy specifically, it directs attention to the political, social, economic and institutional sub-systems that condition what any given actor can decide, and to the pathways by which change propagates or is absorbed [34,35].

Its signature analytic move is the explicit mapping of sub-systems, actors, incentives and feedback relations prior to deciding who should participate and in what. For co-creation this matters because it converts the question of stakeholder selection from a matter of convention or convenience into an analytic one: the actors who must be present are those who hold decision rights or resources within the sub-systems implicated in the problem. A comprehensive assessment of political, social and economic systems also improves an organization’s ability to anticipate the outcomes of its interventions and to align programmes with the priorities of the communities concerned [30]. APHRC has adopted systems thinking as a standard in programme planning and implementation [21].

#### Critical realism and realist evaluation.

One theoretical approach that has gained traction in the context of co-creation in research is critical realism. This perspective emphasizes the importance of iteratively building theory and evidence to address complex social phenomena in public health settings [36]. In acknowledging the interplay between social structures and individual agencies, critical realism provides a robust framework for exploring how co-creation can be effectively implemented to achieve desired health outcomes.

Critical realism is an ontological and epistemological position rather than a method. It holds that a reality exists independently of our knowledge of it, that this reality is stratified the events we observe are generated by mechanisms that are not themselves directly observable and that explanation therefore consists in identifying the mechanisms capable of producing an observed outcome, and the conditions under which they are activated [37,38]. Its account of the relation between social structures and individual agency, in which each condition but does not determine the other, is what makes it attractive to research on collective practices such as co-creation [39].

Its signature analytic move is retrodiction: reasoning back from an observed outcome to the mechanisms and contextual conditions that could have generated it. In applied health policy research this move is most fully developed in the realist evaluation tradition, which operationalizes it through context–mechanism–outcome configurations and asks not whether an intervention works but what works, for whom, in what circumstances and why [40,41]. That tradition has been widely taken up in health policy and systems research, along with a candid literature on the difficulty of applying it consistently [42,43].

We distinguish the two deliberately, since the previous version of this paper did not. Critical realism supplies the philosophical warrant for treating co-created knowledge as a route to explanation and not only to relevance, and recent work has begun to specify what its principles imply for co-creation research in public health [36]. But what our cases actually do reasoning from context and mechanism toward outcomes in policy settings is realist evaluation in practice, and it is more accurate.

#### An integrated analytical framework.

These three frameworks are best understood as complementary rather than competing, since they operate at different levels and address different questions. Systems thinking functions as a macro-level lens on the interdependent political, social, and economic sub-systems within which co-creation occurs; participatory design is principally a methodological commitment governing who participates and how; and critical realism is an epistemological stance concerned with iteratively building explanation of the underlying mechanisms that generate outcomes. Each carries characteristic strengths and limitations. System thinking captures interdependence and context but is analytically demanding and can resist clear decision points; participatory design secures relevance and legitimacy but is vulnerable to the very power asymmetries it seeks to dissolve; and critical realism offers explanatory depth but provides limited prescriptive or operational guidance. For this reason, APHRC typically deploys the frameworks in combination rather than relying on any one of them, and the limitations of each partly account for the governance, stakeholder, and methodological challenges discussed later in this paper. In the cases that follow, we indicate which framework or frameworks each initiative draws upon and how its signature analytic move was operationalized. Because co-creation is a matter of degree, we score each dimension in each case on a three-point scale: substantive, where the dimension was realized with formal standing and consequence for decisions; partial, where it was realized in some phases or for some actors only; and limited, where it was nominal or absent.

#### The second axis: Reach along the research–policy–advocacy interface.

Depth alone does not capture what varies across co-created work. A second axis concerns reach: how far along the research-to-policy-to-advocacy interface co-creation is sustained. We treat that interface not as a linear pipeline in which findings are passed downstream, but as three domains of activity the production of knowledge, engagement with policy processes, and advocacy which co-creation may span to a greater or lesser extent, and between which evidence, priorities and authority move in both directions.

The distinction is not academic. A project may be deeply co-created at the point of knowledge production and then handed over, intact and unexamined, to actors who conduct the policy engagement and advocacy on their own terms. Such a project is co-created research culminating in conventional advocacy and describing it simply as co-creation obscures the point at which the practice stopped. We therefore record for each case not only which segments were reached, but where the transitions occurred and whether the transition itself was co-created, that is, whether the actors who produced the knowledge had any standing in deciding how it would be used.

The two axes are related in a way that clarifies both. The segments of the interface make different demands on the four dimensions. Joint knowledge production is what the research segment requires. Shared agenda setting is the entry condition for the policy segment, since evidence whose questions were framed without policy actors seldom arrives at a live decision. Power sharing and community leadership are what render the advocacy segment legitimate and durable, because advocacy conducted on behalf of a constituency is a different practice from advocacy conducted by it, and only the second survives the end of a grant. On this reading, a project that is strong on knowledge and weak on control is not inconsistent; it is co-created at one end of the interface and not the other. Fig 1 sets out the stages, decision points, dimensions and actor roles across the interface, together with the three questions we use to test whether a transition was itself co-created.

**Fig 1:**
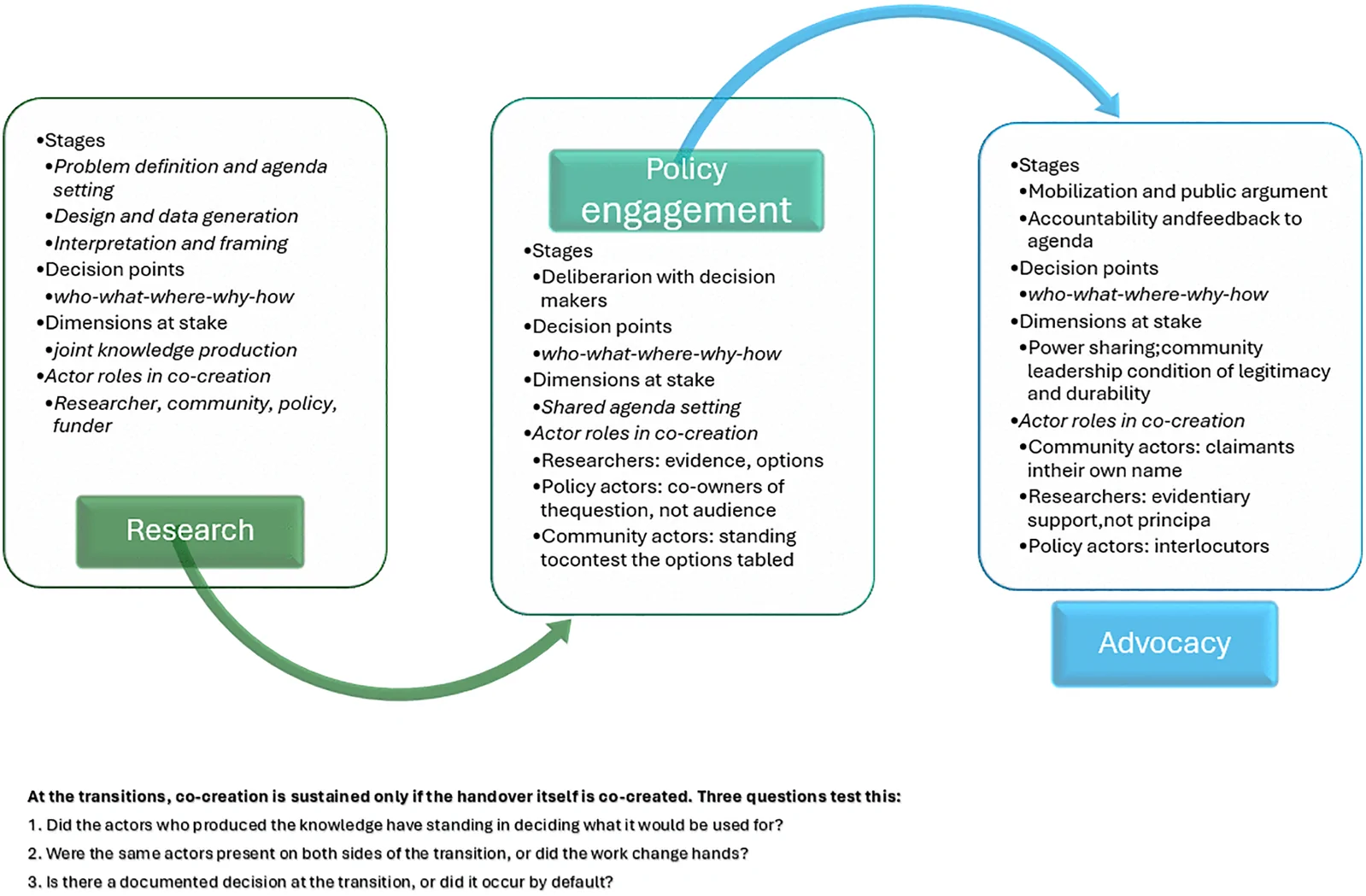
Co-creation across the research–policy–advocacy interface: stages, decision points and actor roles.

We emphasize that six purposively selected cases from a single institution can display a pattern but cannot establish an association, and we draw the corresponding inferences with that constraint in view.

## Co-creation in operation: Tools and methods

The practical means by which the Center conducts co-creation can be grouped under the dimensions they principally serve. We present these here so the cases can be compared to familiar patterns, instead of treating each one as completely unique.

### Instruments servicing shared agenda setting

Agenda setting is co-created through convenings designed to produce a jointly owned research agenda rather than to consult on one: regional and sub-regional stakeholder workshops, partner forums, and standing convenings with education and health bodies [44,45]. The distinguishing feature is that the output is an agenda to which the convened actors have a claim, rather than a report of their views. The co-created regional research agenda for adolescent sexual and reproductive health is the fullest instance [46]. Targeted training in research methods accompanies these convenings, on the reasoning that actors cannot exercise standing over a research agenda without a working grasp of what research can and cannot establish [47]

### Instruments serving joint knowledge production

Knowledge is produced jointly through participatory study designs, the return of preliminary interpretations to participants for correction, and analytic collaboration with policy actors whose accounts of their own incentives are treated as evidence rather than as data to be interpreted around. Projects designed to bridge researchers and policymakers for example work on maternal and child health in Tanzania, which sought to establish a common language among stakeholders belong here [48]. Evidence-informed decision-making is the intended product: public health policy that reflects tangible research outcomes, rather than research that reflects policy already settled [47].

### Instruments serving power sharing, community leadership and the movement of evidence into action

The Center maintains a dedicated policy engagement and advocacy capability, tailored policy-engagement strategies aligned to community priorities [17], and an evidence-to-action model applied in domains including maternal and child health, domestic financing for immunization, and the integration of non-sewered sanitation into national fecal waste management strategies [49,50]. Community inclusion in research uptake particularly of young people, who are the intended users of youth-friendly services is intended to create demand for proven interventions and to establish community-level ownership and accountability through designated representatives [2,46,51]We note, and examine in the discussion, that these instruments distribute standing and visibility more readily than they distribute control.

### Assessing effectiveness

We write about assessing rather than measuring effectiveness, and the distinction is not merely terminological. To measure is to return a determinate quantity on a defined metric. To assess is to exercise judgement against stated criteria, which is what a graded scale of substantive, partial and limited involves, and what claim that co-creation is a matter of degree requires. A paper that argues for gradation while promising measurement is at odds with itself, and the previous version of this one was.

The distinction also permits a more precise statement of what can and cannot be established. Outcomes are frequently measurable: a policy is adopted or it is not; a cohort completes doctoral training, or it does not; a movement exists or it does not. What is not measurable, and must be assessed, is the quality and degree of the co-creation that contributed to those outcomes. We suggest that conflating the two is one reason the field’s evidence about its own effectiveness remains thin on what outcomes are measured and co-creation quality is inferred from them, when the two require different warrants, and a well-conducted co-created project may fail in a hostile policy environment while a poorly co-created one succeeds in a favorable one.

Assessment is accordingly criterion-referenced rather than metric and proceeds along with the two axes: the depth with which each dimension was realized, and the reach across the interface over which co-creation was sustained. The criteria themselves are not universal. Co-creation is not a one-size-fits-all approach [2], and what counts as substantive community leadership differs between a study with incarcerated participants and a youth-led food-rights movement; assessment must be made against what was possible and defensible in the setting, which is why we state the trade-offs case by case rather than scoring in the abstract.

Community engagement adapted to local conditions and culture remains central to any such assessment [52], and the literature suggests that tailored engagement yields substantial benefits for global health projects [52,53]. But engagement is a condition of effective co-creation rather than a measure of it, and we treat it as one of the things to be assessed rather than as the assessment itself. The specific outcome criteria we apply, and the status of evidence supporting them, are set out in our methods.

### Methods of the synthesis

#### Genre and design.

This paper is a reflective, experience-based synthesis of APHRC’s institutional practice rather than a systematic review; it draws lessons from organizational experience to illustrate and interrogate the application of co-creation. The illustrative cases were selected purposively, against four criteria: that, together, they cover the three theoretical frameworks introduced below; that they span APHRC’s principal thematic and methodological domains; that they range across the research-to-policy-to-advocacy continuum; and that they include both long-standing and recent initiatives [53]. A further inclusion criterion was that each case demonstrably exhibits the dimensions of co-creation defined above, rather than partnership or stakeholder engagement alone [14,17–20]. The synthesis draws on a combination of peer-reviewed publications, APHRC institutional and project reports, program documentation, and the authors’ direct involvement in and reflection on several of the initiatives described. Lessons were identified by examining each case against the three frameworks and the four dimensions of co-creation, and then distilling the principles recurring across cases, which are presented in the concluding section. We note the authors’ positionality: as APHRC staff writing about their own institution, we have sought to maintain a reflexive and critical stance, and we return in the Limitations to the constraints this approach places on attribution, generalizability, and reproducibility.

#### Case selection.

Six cases were selected purposively against five criteria: that together they cover the three frameworks set out above; that they span the Center’s principal thematic and methodological domains; that they range across the research-to-policy-to-advocacy interface; that they include both long-standing and recent initiatives [50,53]; and that each demonstrably exhibits the four dimensions of co-creation to some degree, rather than partnership or stakeholder engagement alone [14,17–20]. We note the consequence of the last criterion plainly: it selects cases in which co-creation was present and therefore does not produce a case set capable of testing when co-creation fails. The set is illustrative and analytically motivated, not representative, and no claim of typicality is made for it.

#### Evidence base.

For each case we assembled the available documentary record peer-reviewed publications, project reports, workshop and convening reports, agenda and strategy documents, and public institutional communications and supplemented it with the authors’ direct knowledge where one or more of us was involved. The sources consulted for each case, and their public availability, are listed in S1 Table. No primary data were collected for this synthesis: no partner institution, participant or community member was asked to comment on our account of work in which they took part. Where a claim rests on the author’s recollection rather than on a document, the text says so; where the only available record is an internal document, we indicate this so that readers can weigh it accordingly.

#### Criteria for judging effectiveness and state of evidence.

We distinguish two questions that are frequently conflated: how well an initiative was co-created, and what it achieved. The first is assessed against the four dimensions and the two axes, on the graded scale set out in the introduction. The second requires evidence of a different kind, and we state the criteria we apply. An initiative is treated as having produced a policy outcome where a named instrument was formulated or adopted and the co-created content is identifiable in its text or process record; a research-agenda outcome where a jointly set agenda was published and subsequently drawn upon; a capacity outcome where durable capability was created, evidenced by fellows supported, completions, participating institutions and retention in the region; a partnership outcome where collaboration persisted across more than one funding cycle and produced joint outputs in each; and a constituency outcome where a structure exists that acts in its own name independently of the initiating institution. We do not treat activity, output volume or dissemination as evidence of effect. Table 2 sets out these criteria and what is available for each.

**Table 2:** Criteria for judging effectiveness, distinguished from criteria for assessing co-creation quality.

Criterion	What counts as evidence	Available for this paper	Evidence type
Policy formulation or adoption	A named instrument formulated or adopted, with the co-created content identifiable in its text or process record	Kenyan National Sanitation Management Policy process	Qualitative, with a checkable documentary element
Research-agenda uptake	A jointly set agenda published, and evidence of subsequent use by other actors	Published regional ASRH agenda [2]; uptake not yet assessed	Qualitative
Capacity outcomes	Fellows supported, completions, participating institutions, retention in the region	Held by the Center	Quantitative
Sustained partnership	Collaboration persisting across more than one funding cycle, with joint outputs in each	Guttmacher collaboration across multiple projects and years [20]	Qualitative, documented
Constituency capability	A structure acting in its own name independently of the initiating institution	Zero Hunger Movement [27]; co-owned regional research agenda [2]	Qualitative, partly internal
Community satisfaction or perceived benefit	Systematically collected report from participants or communities	None. Not collected at the time or since	Absent disclosed as a limitation
Funding allocations, service coverage	Attributable change in budget lines or coverage statistics	Not reported; attribution not defensible under this design	Excluded rather than estimated

We report no evidence against a sixth criterion that would properly belong in this list. No systematic account was collected of how participants and communities themselves regarded these initiatives whether they considered the process fair, the outcomes useful, or their own role meaningful. Given that community leadership and ownership is one of our four dimensions, and that we score cases against it, the absence of community-reported evidence is a limitation of consequence rather than a gap to be noted in passing. Our scoring of that dimension rests on documented roles and decision rights, which is a reasonable proxy for standing but not for experience, and we invite readers to weigh it accordingly.

Because the evidence underpinning this synthesis is of uneven strength, we mark it. Each substantive claim is characterized by source publicly documented, where a peer-reviewed publication or public report supports it; internal, where the record is an institutional document not in the public domain; or recollection, where it rests on the authors’ direct experience without a documentary record and by type, as quantitative, qualitative or anecdotal. The convention is applied in full in S1 Table and inline where the distinction affects the weight a claim can bear.

#### Assessment procedure.

Each case was assessed against the four dimensions and against its reach along the interface, using the three-point scale. The authors scored each case independently in the first instance. Discrepant scores were discussed against the documentary record and resolved by argument rather than by averaging; where an author had direct involvement in a case, that author’s account was treated as evidence about what occurred but not as authoritative about how it should be scored, on the reasoning that involvement improves knowledge of events and impairs detachment about their quality. Where residual disagreement remained, the more conservative score is recorded. The scores are judgements, not measurements, and we present them so that they can be contested.

#### Positionality.

All three authors (as of the time of the first submission of this manuscript) are employed by the institution described. We have a professional interest in the institution being well regarded, and readers are entitled to weigh our account in that light. Our principal safeguards have been to report the trade-offs and limits of each case, to score the dimensions in a way that records weakness where we found it, and to state throughout what we cannot establish.

#### Feasibility of this design.

It can support description of what was done, documentation of the co-creation dimensions realized in each case, and the identification of patterns across cases that generate hypotheses and transferable principles. It cannot support causal attribution: the outcomes reported were co-produced with many actors over long periods, and we document contribution and plausible pathways rather than establishing that co-creation produced them. It cannot support counterfactual claims about what would have happened otherwise. It cannot support statistical generalization from six purposively selected cases in one institution. And it is not independently verifiable in the manner of a study working from a public dataset, since part of the evidence is internal and part is the authors’ own experience.

## Co-Creation in practice: Six cases

The cases presented below are illustrative rather than exhaustive. They were chosen to span the three frameworks, to cover APHRC’s principal thematic areas, to range across the research-to-policy-to-advocacy continuum, and to include both long-standing and recent initiatives; each was also required to exhibit the dimensions of co-creation defined earlier. For each case we indicate the actors involved, the co-creation process and the dimensions it evidenced, the framework operationalized, and the outcomes and lessons, while acknowledging, where relevant, the challenges encountered, the ways in which decisions and power were negotiated, and the trade-offs involved. Each case is presented in a common structure: the actors involved; the co-creation process; the dimensions evidenced and their degree; the framework operationalized and the specific analytic move by which it was operationalized; the outcomes; and the trade-offs, limits and contested decisions encountered.

Where a case is weak on a dimension we say so, because the variation across cases is itself part of the analysis rather than an embarrassment to it. Table 1 summarizes these mappings across the cases. Table 2 the degree scores; together they form the analytic spine of the paper rather than a convenience summary, and the cross-case synthesis that follows the cases reads across them to derive the paper’s transferable lessons.

The cases are grouped into four domains: evidence generation, policy processes, capacity strengthening and rights-based advocacy. The first, second and fourth correspond to the segments of the research-to-policy-to-advocacy interface; the third is an enabling activity that builds the capability to traverse the interface rather than traversing it. Each case is presented whole, under the domain in which its distinctive contribution lies, and the reach element records where it travels beyond.

### Co-creation in evidence generation

Evidence generation is the domain in which co-creation is most readily achieved and, on our evidence, least consequential for authority. In both cases presented here, joint knowledge production is substantive: analytic categories were derived from participants’ accounts in one, and measurement assumptions were revised in light of regional contextual knowledge in the other. In neither did that depth extend to control. Shared agenda setting is partial in both, since the questions were fixed at proposal or commission stage; power sharing and community leadership are limited or partial throughout; and both cases stop at the first transition, with the use of the evidence decided by others. The pattern is worth stating plainly because it is the most common form of co-creation in health research and the one most often described without qualification: it is possible to co-produce knowledge deeply while leaving every decision about what that knowledge is for exactly where it was.

#### Ethnographic study in Senegal (2021).

*Actors:* APHRC collaborated with Ibis Reproductive Health, a research organization focused on advancing sexual and reproductive health and rights, to conduct an ethnographic study aimed at documenting the lived experiences of women and girls in Senegal who had been accused of infanticide.

*Process and dimensions*: In a context in which abortion is legally restricted, the study documented the lived experiences of women and girls accused of infanticide [54]. Participants were positioned as narrators and interpreters of their own circumstances rather than as data sources: the enquiry was revised in response to what early participants identified as the salient causes of their situations, and preliminary interpretations were returned to participants and local partner organizations for correction before the framing of the advocacy message was settled. Joint knowledge production was accordingly substantive, in that the analytic categories driving the advocacy derived from participants’ own accounts rather than being imposed on them. Shared agenda setting was partial: the broad question was set by APHRC and Ibis at proposal stage, and participants shaped its content rather than its selection. Power sharing and community leadership were limited, because participants were incarcerated or recently released, and formal co-governance would have exposed them to legal and social risk.

*Framework operationalized:* Co-creation was achieved through a participatory approach where researchers captured individual narratives of marginalized women and girls accused of infanticide. This knowledge informed actions that aligned with the necessity of incorporating perspectives of vulnerable participants. To participants and local partners at defined stages of analysis. The sequence was as follows:

- Framing (decision held by APHRC and Ibis). The research question was set at proposal stage: what circumstances lead to accusations of infanticide, and what happens to those accused. Participants did not hold this decision.
- Access and standing (decision held jointly with local partner organizations). Local legal-aid and reproductive-rights organizations determined how participants would be approached and what protections applied, including what would not be asked and what would not be recorded.
- Instrument revision (decision transferred to participants). After initial interviews, the areas of enquiry were revised to follow what early participants identified as salient. This is the first genuine transfer of design authority.
- Interpretation (decision shared with participants and local partners). Preliminary interpretations were returned for correction before the analysis was fixed. The test applied was whether participants could contradict the research team’s reading and have it changed.
- Framing of the advocacy message (decision held by APHRC, Ibis and advocacy specialists). Which elements of the narratives would drive public argument was settled without formal participant standing, on protective grounds. This is the transition at which co-creation in this case ends.
- Deployment (decision held by advocacy specialists). Advocacy tools were developed and used by specialists. Participants were neither claimants nor principals.

*Context, power and mechanism:* Power was transferred at steps 3 and 4 and retained at steps 1, 5 and 6; the pattern is deliberate and, in our judgement, ethically correct, but it means the case is co-created in respect of knowledge and not of authority. The mechanism the resulting advocacy was intended to act on is the interaction between legal restriction, the absence of confidential post-obstetric care and the social penalty attached to concealed pregnancy, which together convert an obstetric event into a criminal one. Whether the advocacy acted on that mechanism was not tested, and we do not claim that it did.

*Reach and transitions*: The case is co-created within the research segment and does not sustain that quality beyond it. Evidence generation was participatory; the subsequent development and deployment of advocacy tools passed to advocacy specialists, and the women whose accounts constituted the evidence had no standing in decisions about how their narratives would be used in public argument. The transition from research to advocacy was therefore a handover rather than a continuation, and the policy segment was named as an aspiration rather than entered.

*Outcomes:* Funded to support dissemination and the development of advocacy tools [13,18], the collaboration produced, beyond the evidence itself, a set of tools used by advocacy specialists to target the structural drivers of infanticide accusations and to improve reintegration after incarceration and policy change is claimed.

*Trade-off and limit:* The advocacy that followed was conducted on behalf of, rather than by, the women whose accounts underpinned it. This is a defensible response to acute vulnerability, but it is not community-led advocacy.

#### Collaboration with the Guttmacher Institute.

*Actors:* APHRC has maintained a long-term collaboration with the Guttmacher Institute (a research and policy organization specializing in sexual and reproductive health and rights), and by extension the expert team of the Guttmacher-Lancet Commission as well as regional researchers and data providers in adolescent sexual and reproductive health (ASRH).

Process and dimensions: This is a long-running institutional collaboration which has included multiple projects focusing on ASRH as well as measuring the incidence of abortions and the quality post-abortion care of in SSA. This partnership was particularly significant during the Guttmacher-Lancet Commission project (2016–2018), where APHRC provided research support to a global team of experts aimed at shaping a new agenda for sexual and reproductive health and rights [55]. Joint knowledge production was substantive measurement approaches were iteratively refined as successive rounds of estimation exposed the limits of prior assumptions in the region’s settings, with APHRC supplying the contextual knowledge that determined which assumptions were untenable. Shared agenda setting was partial the Commission’s terms of reference were set globally, while regional priorities shaped what the sub-Saharan evidence base contributed. Power sharing was partial and asymmetric in a way worth naming, APHRC’s contribution was evidentiary and intellectual rather than agenda-defining at the outset. Community leadership was limited; this is a partnership between research institutions, and community actors were not principals in it.

*Framework operationalized:* Realist analysis, operationalized through retrodiction, in the following sequence.

- Identifying the anomaly. Recorded abortion incidence and post-abortion care volumes were inconsistent with what service data and legal context implied an observed pattern requiring explanation rather than a quantity to be reported.
- Proposing candidate mechanisms. Legal restriction, provider risk aversion, misclassification of procedures in records, and the cost and distance structure of care were advanced as mechanisms capable of generating the pattern.
- Specifying contextual conditions (APHRC’s principal contribution). Which mechanisms could operate, and where, depended on legal regime, service configuration and social sanction.
- Revising measurements considering the mechanisms, so that estimation did not presuppose recording behavior the mechanisms predicted would be absent.
- Testing implications across settings in which the mechanisms were expected to operate differently, providing a partial check on the explanation.
- Carrying the explanation into normative argument, at which point the work passed to a global coalition in which APHRC participated without setting terms.

*Reach and transitions:* Co-creation was sustained in the research segment and became partial thereafter. The Commission is a global agenda-setting process, and APHRC entered it with evidentiary rather than convening authority. There is no evidence of feedback from that advocacy into APHRC’s subsequent research agenda. This case shows that reach along the interface is not by itself a measure of co-creation: work can travel a long way toward policy and advocacy while the co-creation attenuates at every step.

*Outcomes:* Evidence and analysis contributed to the Commission’s agenda for sexual and reproductive health and rights and to a strengthened regional evidence base on adolescent health [20,21].

*Trade-off and limit*: We retain this case deliberately as the one sitting closest to the boundary of our own definition. It is a genuine collaboration between institutions of distinct standing, and against our four dimensions it is co-creation in a weaker sense than the food-security or capacity-strengthening cases. Its value here is precisely that it marks where partnership shades into co-creation, and it is the clearest instance in our set of the North–South asymmetry elaborated on in the discussion section

### Co-creation in policy processes

Co-creation in policy processes proceeds with those who hold decision rights, and both its strength and weakness follow from that. Because policy actors participate as principals rather than as an audience, the transition from research to policy engagement is not seamless, and co-creation is sustained across both segments; the focus is on the actors it characterizes, who are able to dispute it. But the same logic that identifies the right participants, that those who must be present are those holding decision rights within the implicated sub-systems excludes those who have no decision-making power. This domain therefore produces the inverse of the pattern found in evidence generation, i.e., a reach into policy without community authority.

#### Political economy analyses Kenya’s sanitation policy (2020).

*Actors:* APHRC; national ministries and regulators; county governments; utilities and private and informal service providers; development partners; civil society organizations active in sanitation.

*Process and dimensions:* In 2020, APHRC undertook a problem-based political economy analysis (PBEA) to explore the processes involved in developing Kenya’s National Sanitation Management Policy. This report detailed the key actors and mechanisms involved, thereby providing valuable insights into how policies can be effectively formulated and implemented [56]. Shared agenda setting was substantive, in that sector and policy actors participated in defining what the analysis needed to explain rather than receiving its conclusions. Joint knowledge production was substantive: policy actors’ accounts of their own incentives were treated as evidence rather than as material to be interpreted around them, and the analysis was returned to them for dispute. Power sharing was partial, since the analysis remained APHRC’s output and its funding was institutionally held. Community leadership was limited: those most affected by sanitation policy held few formal decision rights and were correspondingly absent.

*Framework operationalized:* Systems thinking, combined with participatory design, in the following sequence.

- Bounding the system (decision held by the research team with sector informants). The unit of analysis was set as the policy formulation process rather than the sanitation service system, which determined that the relevant actors were those with standing in the policy process.
- Actor and institutional mapping (jointly conducted). Ministries, regulators, county governments, utilities, private and informal providers, development partners and civil society were identified with their formal mandates and the points at which each could advance or block the policy.
- Incentive and interest analysis (jointly conducted). For each actor, the interests served by existing arrangements were characterized, including where non-sewered sanitation threatened established revenue or mandate.
- Identifying leverage and blockage (deliberative, with policy actors present). Points at which the process had previously stalled were traced to specific institutional relations rather than to capacity or awareness deficits.
- Returning the analysis to the actors analyzed (shared decision). The account was presented to participants in the process, who were able to dispute the characterization of their own incentives. This is what distinguishes the exercise from a political economy analysis conducted on a policy process.
- Use (held by policy actors). The analysis informed the continuing formulation process, in which the same actors were principals.

*Reach and transitions:* This case is unusual in beginning at the policy segment rather than arriving at it: the object of enquiry was itself a policy formulation process. Co-creation was sustained across the research and policy segments, and the transition between them was not a handover at all, since the same actors occupied both. The advocacy segment was not entered.

*Outcomes*: The analysis documented the formulation journey of the National Sanitation Management Policy and informed the continuing process [56]. Related work analyzed policy gaps in fecal waste management across Eastern Africa and supported the integration of non-sewered options into national strategies [49].

*Trade-off and limit:* The systems lens converted the question of who should participate from a matter of convention into an analytic result. Its limit is equally clear: participants were those holding decision rights, and communities affected by sanitation policy hold few. This is how a case can score well on reach across two segments while scoring poorly on community leadership.

### Co-creation in capacity strengthening

Capacity strengthening is the only domain in our set in which co-creation operates at institutional rather than project scale, and the only one in which partners hold formal standing in the governance of what is created. Its three linked initiatives form a sequence rather than a set: a fellowship providing skills and partial funding to individual researchers; a consortium that built a doctoral training model with partner universities as principals rather than hosts; and the platforms that followed, evaluated through a process co-created with their founding partners. The pattern is one of deepening institutionalization, and it is where power sharing in our set is most substantive. It also sits differently on the interface: capacity strengthening does not traverse the research-to-policy-to-advocacy span but builds the capability to traverse it.

#### ADDRF, CARTA and the platforms that followed.

*Actors:* APHRC; African universities and research institutions, including Makerere University, the University of Nairobi, the University of Ibadan, Obafemi Awolowo University, the University of the Witwatersrand and the Ifakara Health Institute; non-African partner institutions; doctoral fellows and their supervisors.

*Process and dimensions:* Drawing from the experience and know-how of a large in-house pool of researchers; in addition to a vast network of actors in academia across SSA. The APHRC successfully co-created programs geared towards producing *a critical mass of African researchers leading afro-centric multi-disciplinary research that respond to critical gaps in public and population health within SSA*. The co-creation was institutional and took place through standing structures rather than consultation exercises, includes the convening of key events; including the **Joint Advanced Seminar** for African Researchers, a **Partners’ forum** and a **doctoral supervisor convening** consisting of African universities participating as principals in designing the model they would host [18]. Shared agenda setting and power sharing were accordingly substantive, since partner institutions held formal standing in the governance of the training model rather than participating in a programme designed elsewhere. Joint knowledge production was partial, the co-created object being a training system rather than a body of evidence. Community leadership is partial and differently constituted: the relevant community is the African academic community, which does hold ownership, rather than a population community.

*Framework operationalized:* Participatory design, operationalized through the placement of programme design decisions within partner universities; combined with systems thinking, in the deliberate choice to work with the existing institutional sub-systems of African tertiary education rather than to substitute for them. Rather than replace the prevailing model of doctoral training, CARTA set out to build on existing infrastructure across sub-Saharan African institutions [8].

*Reach and transitions:* This case relates to the interface differently from the others, and we flag the difference rather than force the comparison: capacity strengthening does not traverse the interface but builds the capability to traverse it, its outputs being researchers and institutions rather than evidence, engagement or campaigns. Its position on this axis is best described as enabling.

*Outcomes:* APHRC began as a fellowship programme and now functions as a networking hub for research and capacity strengthening. The African doctoral dissertation research fellowship (ADDRF) stands out as the premier program co-created specifically to aid African academic researchers fulfil their research goals by providing the requisite skills and partial monetary support [22,57]. This endeavor led to the next iteration that is essentially a confluence of African research cohorts supported through a co-created model of doctoral training titled the Consortium of Advance Research Training in Africa (CARTA) [18].; All these in turn gave rise to research platforms such as the AFRICA Research connect and the APHRC virtual academy who support researchers at all levels while learning from a carefully curated evaluation process co-created by their founders [20,58].

*Trade-off and limit:* The time horizon is long the trajectory from fellowship programme to consortium spans two decades which makes attribution difficult and sits awkwardly with prevailing funding cycles. And although partner universities co-own the model, the consortium remains dependent on external funding, so the most substantively power-shared case in our set is also among the least financially self-determining.

### Co-creation in rights-based advocacy

Rights-based advocacy is the only domain in which community leadership is substantive, and the differences between it and the other three are instructive. Both cases span all three segments of the interface. In both, the transitions were not handovers, because the same actors were present on either side. Both show advocacy feeding back into the research agenda rather than only receiving its products, which is the reciprocal relation and can evidence nowhere else. And in both, what was produced was a durable structure with its own membership, a co-owned regional research agenda in one case, a youth-led movement in the other. Rights framing appears to matter here for a specific reason: rights are held by claimants, and a claim is made in the claimant’s own name, so framing itself resists advocacy conducted on a constituency’s behalf. Whether these structures outlast project and fellowship funding is not yet established.

#### Advocacy for adolescent sexual and reproductive health.

*Actors*: APHRC; adolescents and youth-led organizations; national policymakers and parliamentary committees; civil society organizations; regional education and health bodies.

*Process and dimension*: In recognizing the poor adolescent sexual and reproductive health indicators in sub-Saharan Africa, and the research addressing them has been unevenly distributed and only partly African-led, APHRC has focused on key advocacy goals [59]. Rather than set an agenda internally and seek endorsement for it, APHRC convened policy actors, civil society organizations and adolescents to determine jointly which questions warranted regional research investment, producing a co-created regional research agenda for evidence-informed policy and advocacy [2]. Shared agenda setting was therefore substantive and is the defining feature of this case. Joint knowledge production was likewise substantive, since the resulting studies were framed around questions these actors had identified [13]. Power sharing was partial: the agenda was jointly owned, but funding, convening authority and publication platforms remained institutional. Community leadership was partial adolescents shaped priorities and participated in advocacy but did not lead it.

*Framework operationalized*: Participatory design, operationalized through the transfer of agenda-setting authority to non-academic actors before any study was designed; combined with systems thinking, which identified the education, health and legislative sub-systems that would have to move together for advocacy on comprehensive sexuality education and child marriage to succeed.

*Reach and transitions:* This is one of two cases in which co-creation is sustained across all three segments, and the clearest evidence in our set for the feedback posited earlier: because the research agenda was itself co-created with policy and advocacy actors [2], advocacy priorities determined what was researched rather than only receiving what research produced. Since the agenda-setting actors and the advocacy actors were largely the same, there was no handover to lose.

*Outcomes:* A co-created regional research agenda [2]; sustained advocacy for access to education and comprehensive sexuality education, for legal reform to end child marriage, and for universal access to reproductive health services [2,22].

*Trade-off and limit.* Agenda ownership was shared while resource and platform control was not, and this asymmetry sets a ceiling on how far the work can be described as adolescent-led. Advocacy on these issues is also politically contested, and the negotiation of what could be argued publicly, and by whom, was not itself an open or jointly governed process.

#### Food security and the right to proper nutrition.

*Actors:* APHRC; national and county government officials; civil society organizations; grassroots and youth-led groups in urban poor communities; residents of informal settlements in Nairobi and elsewhere; academic partners in Kenya and South Africa.

*Process and dimensions:* The Center has worked in advancing the right to food across Africa through collaborative, community-centered initiatives. In April 2024, APHRC co-hosted a multi-stakeholder dialogue in Kisumu, Kenya, uniting over 130 participants including government officials, civil society, and grassroots organizations to advocate for rights-based food policies and accountability mechanisms aimed at achieving Zero Hunger by 2030 [60]. Complementing this, APHRC’s Zero Hunger Initiative has supported urban poor communities in Kenya to establish the Zero Hunger Movement, empowering youth-led groups to raise awareness and hold duty bearers accountable for realizing the right to food [61]. Additionally, through a *Wellcome (Formerly known as Wellcome Trust)* a global research foundation fellowship, APHRC engaged urban populations to deepen public understanding of constitutional food rights and explore strategies for their fulfillment [50,62]. All four dimensions are substantive, i.e., agendas were set with communities and civil society, lived experience was privileged and published as such, community organizations hold standing in the convening structures, and the movement acts in its own name.

*Framework operationalized:* Systems thinking combined with participatory action design, applied to the food system as a set of interdependent policy, market and community sub-systems, with a rights-based framing that identifies duty bearers and claimants rather than beneficiaries and providers.

Reach and transitions. This is the case that reaches furthest on both axes. Research on lived experience and on rights-based approaches fed a multi-stakeholder policy dialogue convening government, civil society and grassroots organizations, which in turn supported a youth-led movement conducting accountability advocacy in its own name. Co-creation is sustained across all three segments, and the transitions were not handovers, since community and civil society actors were present on both sides of each. Critically, the advocacy is conducted by the constituency rather than on its behalf, which is what distinguishes this case from the Senegal study at the far end of the interface.

*Outcomes:* A multi-stakeholder dialogue with documented participation; a constituency-led movement conducting independent accountability work; published research on human rights-based approaches to food security in Kenya and South Africa emphasizing inclusive and equitable food systems [50,62].

*Trade-off and limit:* Outcomes in this domain are the hardest in our set to attribute, since food policy moves slowly and many actors are involved. The open question is durability: whether a constituency-led structure supported through project and fellowship funding remains active once that funding ends is not yet established, and we do not claim that it will.

### What the cases show when read together

Read individually, the cases illustrate epileptic evidence towards sustainable co-creation across domains. Read across Tables 3 and 4, they support six observations that no single case could sustain.

**Table 3:** The six cases: domain, framework and signature move, actors, outcome and principal trade-off.

Case	Domain	Framework and signature move	Key actors	Principal output/ outcome	Principal trade-off or limit
Senegal ethnography (2021)	Evidence generation	Participatory design transfer of interpretive authority over narrative meaning	APHRC; Ibis; women and girls accused of infanticide; legal-aid organizations	Advocacy tools targeting drivers of infanticide accusations and reintegration	Advocacy on behalf of, not by, participants; protection constrained power sharing
Guttmacher collaboration	Evidence generation	Realist analysis retrodiction from incidence and care-quality patterns to generative mechanisms	APHRC; Guttmacher Institute; Commission expert team	Contribution to the Commission’s SRHR agenda; strengthened regional evidence base	Boundary case; agenda set globally; clearest North–South asymmetry in the set
Kenyan sanitation analysis (2020)	Policy processes	Systems thinking mapping of actors, incentives and sub-systems; with participatory design	APHRC; ministries and regulators; county governments; providers; civil society	Analysis informing the National Sanitation Management Policy process	Policy-actor centered; affected communities largely absent
ADDRF, CARTA and platforms	Capacity strengthening	Participatory design with systems thinking building on existing university sub-systems; partner governance	APHRC; African and partner universities; doctoral fellows	Co-created doctoral training model; research-networking platforms	Long horizons; continued donor dependence for a structure otherwise co-owned
Adolescent SRH advocacy	Rights-based advocacy	Participatory design with systems thinking agenda-setting authority transferred before study design	APHRC; adolescents; policymakers; civil society organizations	Co-created regional research agenda; advocacy for CSE and against child marriage	Agenda co-owned but resources and platform control retained institutionally
Right to food	Rights-based advocacy	Systems thinking with participatory action design rights framing identifying duty bearers and claimants	APHRC; government; civil society; grassroots and youth-led groups	Multi-stakeholder dialogue; Zero Hunger Movement	Outcomes hardest to attribute; durability beyond project funding untested

**Table 4:** Depth and reach of co-creation.

Case	Shared agenda setting	Joint knowledge production	Power sharing	Community leadership	Research	Policy engagement	Advocacy	Feedback to agenda
Senegal ethnography	P	S	L	L	S	N	P	N
Guttmacher collaboration	P	S	P	L	S	P	P	N
Kenyan sanitation analysis	S	S	P	L	S	S	N	N
Capacity strengthening (ADDRF, CARTA)*	S	P	S	P	S	P	N	P
Adolescent SRH advocacy	S	S	P	P	S	S	S	Y
Right to food	S	S	S	S	S	S	S	Y

Key: Depth: S = substantive, P = partial, L = limited. Reach: S = co-creation sustained in this segment, P = segment entered but co-creation partial, N = segment not entered. Feedback: whether advocacy or policy engagement dem.

* Capacity strengthening operates as an enabling condition for the interface rather than as a traverse of it; the reach scores indicate the segments its outputs directly served.

First, the dimensions are unevenly realized, and the unevenness is patterned rather than random. Joint knowledge production is substantive in five of six cases; shared agenda setting in four; power sharing in two; community leadership and ownership in one. Co-creation in our practice has been strongest where knowledge is made and weakest where control is held. This is the central self-critical finding of this synthesis, and it gives concrete form to the concern, in our discussion, that participation can be substantive in method while remaining thin in authority.

Second, the two cases in which power sharing and community ownership are substantive CARTA and the Zero Hunger Movement share a feature the others lack: in each, the co-created output was a durable structure with its own governance and membership, rather than a project deliverable. Where co-creation produces an institution, ownership can outlive the funding cycle; where it produces a report or a tool, it generally does not. The choice of output form is therefore itself a power-sharing decision, and one usually made early and implicitly.

Third, framework combination is a response to failure modes rather than a rhetorical preference. The two cases operationalizing a single framework Senegal and Guttmacher are the cases weakest on the dimensions that framework does not address: participatory design alone secured interpretive inclusion without altering control, and realist analysis alone secured explanatory depth without altering participation. The cases combining systems thinking with participatory design score more evenly, because the systems lens identifies who holds decision rights and the participatory commitment then places those actors in the process.

Fourth, the weakest cases on community leadership are those whose intended beneficiaries were the most vulnerable or the most legally exposed. The relationship between protection and participation is not simply positive; ethical safeguards can and do constrain power sharing. We do not think this tension can be resolved in general, but it should be stated and reasoned about case by case rather than passed over, and we examine it in later sections.

Fifth, co-creation in our practice does not fail within segments of the interface; it fails at the transitions between them. Every case in our set is co-created in the research segment. What distinguishes them is whether that quality survives the move to policy engagement and again to advocacy, and where it does not, the break occurs at a moment that was neither designed nor evaluated nor recorded evidence is simply passed to those whose business the next segment is. The two cases in which co-creation is sustained throughout are the two in which the same actors were present on both sides of each transition, so that there was no handover to lose; they are also the two cases scoring highest on power sharing and community ownership. The two cases in which co-creation does not survive past the research segment are the two scoring lowest on those dimensions. With six purposively selected cases we can report this pattern but cannot test it, and we offer it as a hypothesis for the systematic evaluation we call for in Section 7.

Sixth, the domains of co-creation differ systematically in the dimensions they are able to realize, and the differences are not accidental. Evidence generation realizes co-creation in knowledge; policy processes realize it in access to those who decide; capacity strengthening realizes it in institutions; and only rights-based advocacy realizes it in authority. Each domain’s characteristic strength is also the source of its characteristic limit: the logic that makes policy-process co-creation effective convening those with decision rights is the same logic that excludes those without them. Institutions selecting a domain in which to work are therefore also selecting, usually without noticing, which dimensions of co-creation will be available to them.

## Discussion

### Comparing participatory action research, realist evaluation and implementation science for co-creation

Three core theories have been presented in this exploration, and our cases stand in a different relation to each. Table 5 summarizes where our practice confirms them, where it nuances them, and where it puts pressure on them.

**Table 5:** Underlying theory confirmations, deviations and contradictions.

Underlying theory	Confirms	Nuances	Contradicts
Participatory action research	That the decisive moment of participation is problem definition, not data collection (co-created regional ASRH research agenda)	Extends co-creation to institutional and regional scale, where the bounded community and the action–reflection cycle the tradition assumes are unavailable (CARTA; multi-country agenda)	The presumption that deeper participation is always preferable; the Senegal case shows protective constraint and power sharing in genuine tension, which the literature under-theories
Realist evaluation	The contingency premise; framework combinations outperform single frameworks in a form resembling a context–mechanism–outcome claim	Co-creation is better treated as a property of the process producing an intervention and its evaluation than as an intervention with mechanisms of its own	If knowledge is genuinely co-produced, whose programme theory is under test? Co-creation destabilizes the standpoint from which retrodiction is performed
Implementation science	That context conditions outcome; the inner/outer setting distinction maps onto the organizational seam identified within the institution studied	Engagement is constitutive of what gets specified, not an implementation strategy applied to something already specified	Fidelity is a difficult criterion for co-created work, where principled adaptation may signal success; and the field rarely asks whether the research-to-implementation handover was itself co-created

On Participatory action research. Our cases confirm the literature’s central contention that the decisive moment of participation is the definition of the problem rather than the collection of data, and that research conducted with rather than on its participants produces different questions and not merely better-received answers [63–65]. The co-created regional research agenda for adolescent sexual and reproductive health is a clean instance [46]. Our cases nuance the application of this theory by showing co-creation operating at institutional and regional scale: participatory action research is characteristically bounded a community, a problem, a cycle of action and reflection and the doctoral training consortium and the multi-country research agenda are neither bounded in that way nor reducible to it. And our cases put pressure on the theory’s emancipatory presumption that deeper participation is always to be preferred. In the Senegal study the participants were incarcerated or recently released and legally exposed, and formal co-governance would have placed them at risk; the ethically defensible choice was to limit their formal role while maximizing their interpretive authority. Literature offers little guidance on when participation should be constrained for protective reasons, and treats such constraint as a failure of ambition rather than as a decision to be reasoned about.

For Realist evaluation. Our cases confirm the premise on which the theory tests and the finding that cases combining systems thinking with participatory design score more evenly than cases operationalizing a single framework is a context–mechanism–outcome claim in its application, as well as in the rigor of its theoretical concept [41,43]. They deviate from the literature by suggesting that co-creation is not well understood as an intervention with mechanisms of its own, but as a property of the process by which an intervention, and the evaluation of it, are produced; if that is right, realist methods should be applied to the co-creation and not only through it, and our two axes are a first proposal for what its “where” and “how” might look like. They also raise a difficulty we cannot resolve; programme theory in realist evaluation is normally specified by the evaluator and tested against the case, but if knowledge is genuinely co-produced it is not obvious whose programme theory is under test, and co-creation destabilizes the standpoint from which retrodiction is performed. We note the problem rather than solve it.

When casting a lens on Implementation science. Our cases confirm the literature’s foundational insistence that context conditions outcome, and its distinction between inner and outer setting maps closely onto our own finding about an organizational integration within the institution studied [66,67]. They may deviate in a way we think has an impact: implementation science has largely treated stakeholder engagement as an implementation strategy and input that improves the adoption of an already specified intervention whereas our cases indicate that engagement is based on what comes to be specified at all. Engagement that begins after specification is a different practice from co-creation; however similar its activities look. And our cases bear on two active debates in the field. Fidelity, which sits near the core of the implementation-outcomes groupings [68], is a difficult criterion for genuinely co-created work, since adaptation by the actors who co-produced an intervention may be evidence that co-creation succeeded rather than that implementation drifted; we offer our cases as a contribution to the existing literature on principled adaptation rather than as identifying a gap. And implementation science studies the implementation of research findings while rarely asking whether the handover from research to implementation was itself co-created, which is exactly where our cases locate the loss.

Read together, this comparison is where we would locate what is distinctive in the present account. Participatory action research is bounded by the project, realist evaluation by the intervention, and implementation science by the implementation phase. None of the three asks how far co-creation is sustained across the full span from research through policy engagement to advocacy, or what becomes of it at the joints between them. That question, and the finding that the joints are where co-creation is most often lost and least often examined, is what this paper offers.

### Power and equity: Who sets agendas, who controls resources, whose knowledge counts

A fuller treatment of power is warranted in any discourse concerned with African-led co-creation; we cannot leave the topic without asking some critical questions. We therefore answer four for each case and report the answers in Table 6. Who sets the agenda: co-creation’s promise is undercut where research questions are settled in advance and community, or policy actors are invited only to ratify them. Who controlled resources: funding is rarely co-owned, and donors frequently retain decisive influence over ostensibly co-created work [69]. Whose knowledge was privileged: the question of epistemic justice central to co-creation’s aspiration to value non-academic knowledge on equal terms [70]. And what remained unresolved, since reporting only the tensions we managed would misrepresent the practice [71].

**Table 6:** Critical case questions on co-creation.

Case	Who sets the agenda	Who controlled resources	Whose knowledge was privileged	What remained unresolved
Senegal ethnography	APHRC and Ibis, at proposal stage	International grant, held by the research institutions	Participants’ accounts in analysis; professional judgement in the advocacy frame	Participants bore disclosure risk while institutions captured outputs; Senegalese partners bore legal exposure without standing in the framing decision
Guttmacher collaboration	Set globally, through the Commission’s terms of reference	Northern partner and its funders	Regional contextual knowledge admitted as input to a globally framed measurement agenda	Contribution recognized, framing authority not; whether regional priorities would have produced a different agenda was never tested
Kenyan sanitation analysis	Jointly with sector and policy actors	Project funds held by APHRC	Policy actors’ accounts of their own incentives, treated as evidence	Those most affected by sanitation policy hold no decision rights and had no route into a process defined by them
Capacity strengthening	Co-set with partner universities	Donor funds channeled through APHRC as consortium lead	Partner universities as principals; standards drawn substantially from northern doctoral norms	Partner institutions co-govern a model they do not finance; the hub role concentrates authority within Africa
Adolescent SRH advocacy	Co-set with adolescents, civil society and policy actors	Institutional; convening and platform control retained	Adolescent and civil society priorities in agenda setting; academic conventions in what counted as evidence thereafter	Who decides what may be publicly argued on contested issues, and who bears the consequences of arguing it
Right to food	Co-set with communities and civil society	Fellowship and project funding, institutionally held	Lived experience of food insecurity, privileged and published as such	Whether the constituency-led structure is sustainable once institutional funding ends

Read across the cases, the answers show a clear divergence between two of these. Agendas were substantively shared in four of six cases and partially shared in the remaining two. Resources were shared in none. Every initiative described in this paper was financed through an instrument held by a single party in most cases us, and in one case a northern partner and its funders and in no case did the actors with whom the agenda was shared hold any claim on the funds that realized it. We take this to be the material condition underlying the pattern reported in our synthesis, that co-creation in our practice is substantive where knowledge is made and weak where authority is held. It also sets a limit on what better process design can achieve: an institution can distribute interpretive authority, agenda-setting authority and even formal governance standing without distributing the capacity to continue if it withdraws.

On whose knowledge is privileged, the pattern is more varied and, in some respects, more encouraging. Lived experience was privileged and published as such in the right-to-food and Senegal cases; adolescent and civil society priorities determined the content of a regional research agenda; policy actors’ accounts of their own incentives were treated as evidence in the Kenyan analysis. What was rarely displaced is the authority to determine what counts as evidence at all, and the disciplinary conventions governing that determination remained academic in every case.

We note, reflexively, that APHRC is not external to these dynamics. As an African-led institution operating within a globally financed health-research system, it is simultaneously subject to asymmetries in relation to Northern funders and partners and a holder of power in relation to the communities and smaller institutions with which it works. Recognizing this dual position, and managing it deliberately, is in our view a condition of genuine rather than nominal co-creation.

One aspect of this dual position is structural and specific to us. APHRC houses policy engagement and advocacy in a dedicated unit, distinct from the research programmes that generate the evidence. The arrangement concentrates genuine specialist capability and is, we think, correct. But it also institutionalizes a transition of the kind that creates a point at which co-produced evidence changes hands, and at which the community and policy actors who helped produce it may have no standing in deciding how it is used. Where such a seam exists by design, the co-creation of the handover itself decides what the evidence is for, and who speaks with it must be designed too, and we do not currently do this systematically.

### Ethical dilemmas and unresolved tensions: The Senegal ethnography examined

We examine one case in detail, because generalized acknowledgements of ethical complexity are cheap and specific ones are not. The Senegal study stands out: it is where the dilemmas were sharpest, and where co-creation most clearly failed to resolve them. The participants were women and girls accused or convicted of infanticide, incarcerated or recently released, in a jurisdiction where abortion is legally restricted. Formal co-governance of the project a participant advisory structure, named co-authorship, public standing in the advocacy would have created a documented link between identifiable individuals and criminal matters, and consent to participate in research is not consent to be publicly identified as an author of a campaign. We limited participants’ formal role for that reason and maximized their interpretive authority instead. We continue to think this was right. The decision to limit who gets involved was made by the people in charge and for them, protection and convenience feel the same. The only check on this is that they must write down their reasons at the time, and we’re allowed to dispute it.

The distribution of benefit and risk. Participants bore the risk of disclosure, of re-traumatization in the telling, and of consequences within their communities. The institutions bore almost none, and received publications, grant outputs, advocacy assets and reputational credit. Consent processes were observed, and this asymmetry survives them. Working together with participants didn’t solve the problem. It just made the information-gathering seem more respectful, but it was still taking from them without giving back. As far as we can tell, there was no way for participants to get anything in return except maybe some vague legal improvements down the road.

Advocacy framing against lived experiences. To be effective, advocacy must use a message that rallies support and that message must be easier to grasp than the complex accounts that back it up. Participants’ own narratives were, as such narratives usually are, more ambivalent than a campaign can accommodate not all described themselves as wronged, and the moral self-understanding of a woman who has been prosecuted is not reducible to the category of victim. Overly simplify a story does not serve the broader group. This dilema was resolved by professional judgement at the point where participants had least standing, and we record it as unresolved rather than as managed.

Unequal exposure among the co-creators. The risks of advocating around a criminalized practice were not shared equally among those conducting it. An international research organization and a Nairobi-headquartered institution carried reputational and, at worst, operational risk. Senegalese partner organizations and individuals carried legal and social risk, in the jurisdiction where the argument was being made and where they would remain after the project closed. Those bearing the greater exposure had less standing in the decision that generated it. We regard this as the most serious equity failure in the case, and it is the one least addressed by any co-creation framework we are aware of, including our own, since such frameworks attend to the relation between researchers and communities and rarely to the differential exposure of the researchers.

Whose community? This case exposes an assumption embedded in co-creation frameworks generally. They presuppose a community with interests sufficiently shared that participation could represent it. Here the women, their families, the neighbors who reported them, the providers who treated them and the officials who prosecuted them do not constitute such a community; participation with one part of it was opposition to another. Where interests genuinely conflict, community participation is a decision about which part of a community to work with and should be described as such rather than as inclusion.

What co-creation did, and did not, do. It changed how the knowledge was made: the analytic categories that drove the advocacy came from participants rather than from us. It did not change who decided what the knowledge was for. We would not conduct the study differently in respect of participant protection. We would design the transition to advocacy differently, record the reasoning behind protective constraints at the time they are imposed, and negotiate the distribution of risk among partners explicitly before rather than after the framing decision.

### Equitable partnerships and the decolonizing debate: Our own position

The questions raised above are the subject of a growing literature on equity in global health research partnerships, which has moved from diagnosis toward instruments: codes of conduct governing research in resource-poor settings, fairness assessment frameworks for research institutions, fair contracting practices, and sustained critique of parachute research and of the authorship arrangements that accompany it [72–75]. A parallel body of work has argued that funders, rather than researchers, are the decisive actors in structuring these inequities, since the instruments through which money move determine who can lead, who must sub-contract, and whose institutional overheads are recognized [76,77]*.* The decolonizing literature supplies the epistemic dimension: the foreign gaze, and the systematic discounting of certain knowers and forms of knowledge [78–80]*.*

We situate our own account within this literature rather than beside it and doing so requires two statements about our position. The first is that APHRC is subject to these asymmetries. Its agenda is shaped by funding instruments it does not control, and the history recounted in the introduction when a funder withdrew is an illustration rather than an exception. The second is that APHRC holds power in these relations too: in relation to the communities it researches, to the smaller institutions with which it partners, and as the entity through which consortium funds flow to universities that co-govern programmes they do not finance.

## Limitations and challenges of co-creation

Co-creation in research, policy engagement, and advocacy for global health presents several obstacles that can impede its effectiveness. Effective collaboration often necessitates overcoming barriers related to communication, differing objectives among stakeholders, and the integration of diverse perspectives into a cohesive strategy [71]. Engaging communities in research efforts, particularly in fields such as One Health, requires careful planning and execution to ensure meaningful participation and input from all relevant parties [71,81].

It is worth stating that the critical literature on co-production is considerably more skeptical than the enthusiasm surrounding the term would suggest, and that this skepticism is common. Oliver, Kothari and Mays in 2019 argued that the costs of co-production in time, in career risk borne unequally by junior and community participants, in the moral hazard of enlisting participants in legitimating research they cannot meaningfully shape may in some circumstances outweigh its benefits, and that the field’s evidence base for its own claims is thin [82]. Additionally Williams and colleagues document how co-production can obscure rather than redistribute power, since the informal relations through which it operates are less visible and less contestable than the formal hierarchies it replaces [83]. We regard these as warranted criticisms rather than counsels of despair, and the reading of our own cases, shows strength on joint knowledge production, but weak on power sharing, and lapsing at the transitions is consistent with them.

### Governance

One of the primary challenges in co-creation is establishing effective governance structures. Maintaining momentum throughout the co-creation process requires clear stage gates or key milestones to review progress and ensure teams are not overburdened with frequent or unnecessary reporting activities [2,71,81]. It is crucial for organizations to define expectations early at these milestones, including progress made, decision-makers, and the scope of decision-making [71]. Additionally, agreements on conditions, rights, and outcomes related to the termination of partnerships can help mitigate risks associated with unfavorable transitions [71].

### Resource allocation

Restrictive funding models often limit organizations’ capacity to adapt to rapidly changing environments and adequately meet stakeholder needs [69]. Inadequate funding can hinder the development and implementation of essential activities and processes, such as decision support tools and costed M&E strategies necessary for effective co-creation [69,84].

### Stakeholder engagement

Engaging stakeholders effectively poses a new set of difficulties. While stakeholder engagement is essential for successful co-creation, it requires a nuanced understanding of the local context and careful planning as well as arbitration (when required) [45,85]. The process involves mapping and prioritizing stakeholders, budgeting for engagement activities, and monitoring their effectiveness. However, the challenges and ethical considerations associated with stakeholder engagement can complicate these efforts [86].

### Methodological

Traditional top-down co-creation approaches often fall short of addressing complex health (and health system) issues due to their failure to account for the dynamic and interdependent nature of health determinants [87]. Despite the potential benefits of co-creation, the methods employed must be thoughtfully considered. Without a methodological framework that supports co-creation, there is a risk of replicating the limitations of traditional approaches, thus preventing the desired outcomes [71,88].

## Scope and reflexivity of this synthesis

As a reflective, single-institution synthesis, this essay carries inherent methodological limitations that bear on its rigor and reproducibility. The illustrative cases were selected purposively rather than systematically, and the account is not intended to be exhaustive or statistically representative of co-creation in the region. It draws in part on internal and grey literature and on the authors’ own involvement in the initiatives described which, while affording access to otherwise undocumented detail, introduces the potential for selection and self-presentation bias and limits independent reproducibility. Relatedly, the paper documents APHRC’s contribution to, and plausible pathways toward, the outcomes discussed rather than establishing causal attribution; many of the outcomes reported are co-produced with numerous actors and unfold over long horizons. Systematic, independent evaluation of the impact of co-creation, and of the conditions under which it succeeds or fails, remains an important avenue for future work.

### Navigating challenges

While co-creation presents many opportunities, it also poses significant challenges that must be addressed proactively. Organizations should be prepared to identify potential obstacles and devise strategies to mitigate them. These challenges may include balancing differing interests, managing conflicts, and navigating ethical considerations in stakeholder engagement [81]. The creation of a collaborative research agenda and communities of practice can also support overcoming these challenges by promoting a culture of shared responsibility and innovation [46,81].

## Conclusions and next steps

The APHRC experience around co-creation is an exemplar that blends the theories into measurable practice with a potential for long-term impact. APHRC has used the concept of cocreation in advocacy-driven initiatives for research uptake on key issues affecting the youth. The center also employed a combination of participatory design and system-thinking approaches in its collaboration with the Guttmacher institute and varied policy actors. With the mindset of influencing policy and processes, the APHRC has used critical realism in developing problem based political economy analyses.

Several transferable lessons emerge across these experiences. First, co-creation is most productive when treated as a disciplined practice with explicit dimensions namely shared agenda setting, joint knowledge production, power sharing, and community leadership rather than as a synonym for collaboration or consultation. Second, the theoretical frameworks are most effective in combination, since each compensates for the others’ limitations. Third, embedding policy and community actors from the problem-definition stage is what renders policy-relevant evidence and lends advocacy legitimacy. Fourth, institutional capacity for co-creation is itself co-created and is necessarily a long-term endeavor, as the trajectory from a fellowship program to the CARTA model illustrates. Fifth, power asymmetries, governance clarity, and funding flexibility must be managed deliberately; where they are not, participation risks becoming tokenistic. These lessons are offered not as features peculiar to APHRC but as principles that other research institutions, funders, and policy actors may adapt to their own settings. Box 1 states them as guidance that can be acted upon. We note that several of its budgeting for transitions, disclosing the structure of resource control to partners, funding structures rather than projects are inferences from our findings rather than practices we have consistently followed ourselves, and we present them as such (Table 7).

**Table 7:** Dimensional and transferable lessons form APHRC.

Area	Guidance
Governance	Specify decision rights rather than levels of participation: for each decision point, name who decides and who may object, before work begins. Agree in advance how disagreement is resolved and who prevails a shared process is what distinguishes co-creation from consultation. Set stage gates at the transitions between research, policy engagement and advocacy, within workstreams. Agree term termination at the outset, including what becomes of jointly owned outputs if a partner withdraws.
Funding	Budget for the transitions: engagement and advocacy costs commonly fall outside research grant lines, which is one reason handovers happen by default. Seek flexibility in scope and not only in timeline co-creation that cannot change the research question in response to partners is consultation. Disclose the structure of resource control to partners at the outset; undisclosed asymmetry is a frequent source of later breakdown. Where ownership is intended to outlast the grant, fund a structure rather than a project.
Community roles	Decide which role is offered by narrator, interpreter, co-analyst, co-decider, principal and say so, since ambiguity is generally resolved in favor of the researchers. Distinguish protective constraint from convenience: where participation is limited for safety, record the reason at the time; where it is limited for cost or speed, do not describe the result as co-creation. State whether advocacy is conducted by a constituency or on its behalf. Give the constituency standing at the framing decision, not only at data collection, since framing determines what the work is for.
Transitions	Test each handover with three questions: did the actors who produced the knowledge have standing in deciding its use; were the same actors present on both sides; is there a documented decision, or did the transition occur by default? Where research and policy engagement are institutionally separated, treat the seam as a designed object with its own governance rather than as an administrative boundary.
Assessment	Assess degree rather than measuring it: score each dimension against stated criteria on a graded scale and publish the scoring so that it can be contested. Separate the measurement of outcomes from the assessment of co-creation quality; a well-co-created project may fail in a hostile environment and a poorly co-created one may succeed in a favorable one. Score reach as well as depth, since a project co-created deeply in one segment is not thereby co-created. Report weak scores; a case set containing no weaknesses is a communications document rather than an analysis.

For APHRC, the next logical priorities include strengthening the measurement of co-creation’s impact, formalizing internal guidance and dimensions for co-creation, and deepening the reflexive evaluation of its own practice. For the wider field, the priorities are threefold: developing a shared understanding of what co-creation means and does not mean; building agreed methods for evaluating co-creation and its outcomes; and advocating for funding models suited to the iterative, relational nature of co-created work. Each of these responds directly to the limitations discussed below, and together they chart an agenda for making co-creation both more rigorous and more genuinely equitable.

## Supporting information

S1 Table Evidence base by case. (DOCX)

S2 Table Candidate impact indicators, for completion from institutional records. (DOCX)
