# Supplementary material for: Co-creation in research, policy engagement and advocacy: Lessons and limits from two decades of practice at the African Population and Health Research Center
Source: PLOS Glob Public Health. 2026 Sep 30;6(9):e0007405. doi: 10.1371/journal.pgph.0007405 (PMC13626369; doi:10.1371/journal.pgph.0007405)
Supplement: Supplementary file 1 [file pgph.0007405.s001.docx]

**S1 Table. Evidence base by case**

| **Case** | **Documentary sources** | **Author involvement** | **Publicly available** |
| --- | --- | --- | --- |
| Senegal ethnography (2021) | Peer-reviewed publication on infanticide in Senegal [18]; regional research-priorities paper [13]; project documentation | **[AUTHORS: partial]** | Partly |
| Kenyan sanitation analysis (2020) | APHRC report on the National Sanitation Management Policy formulation journey [19]; policy-gap analysis on faecal waste management [34] | **[AUTHORS: partial]** | Yes |
| Guttmacher collaboration | Guttmacher–APHRC partnership record [20]; Commission outputs; regional ASRH scoping review [21] | **[AUTHORS: complete]** | Yes |
| Adolescent SRH advocacy | Co-created regional research agenda [2]; research-priorities paper [13]; ASRH scoping review [21]; advocacy strategy [7] | **[AUTHORS: complete]** | Mostly |
| Capacity strengthening | Peer-reviewed reflections on capacity strengthening [9]; ADDRF programme paper [24]; CARTA documentation [8]; platform records [10,25] | **[AUTHORS: partial]** | Mostly |
| Right to food | Lived-experience study [29]; Zero Hunger Initiative documentation [27]; fellowship project record [28]; dialogue report **[VERIFY: source]** | **[AUTHORS: partial]** | Partly |
