# Supplementary material for: Co-creation in research, policy engagement and advocacy: Lessons and limits from two decades of practice at the African Population and Health Research Center
Source: PLOS Glob Public Health. 2026 Sep 30;6(9):e0007405. doi: 10.1371/journal.pgph.0007405 (PMC13626369; doi:10.1371/journal.pgph.0007405)
Supplement: Supplementary file 2 [file pgph.0007405.s002.docx]

**S2 Table. Candidate impact indicators, for completion from institutional records.**

| **Case** | **Indicator** | **Status** |
| --- | --- | --- |
| Kenyan sanitation analysis | Whether the National Sanitation Management Policy was adopted; whether non-sewered options appear in the adopted text; date and instrument reference | O public documentary |
| Capacity strengthening | Fellows supported by programme and cohort; doctoral completions; participating institutions; proportion retained in African institutions post-award | O held internally |
| Right to food | Participants and organizations at the Kisumu dialogue by category; constituent groups in the Zero Hunger Movement; activities conducted independently of APHRC | O in part |
| Adolescent SRH advocacy | Publication of the co-created regional agenda; studies subsequently conducted against it; citation or adoption by national or regional bodies | O in part |
| Guttmacher collaboration | Number of joint projects and years spanned; Commission outputs to which APHRC contributed | O public record |
| Senegal ethnography | Advocacy tools produced; organizations using them; any legal or policy change subsequently enacted | O in part policy change not established and should not be implied |
| All cases | Changes in funding allocation or service coverage | N excluded |

Key: O = obtainable and verifiable; N = not attributable under this design.
